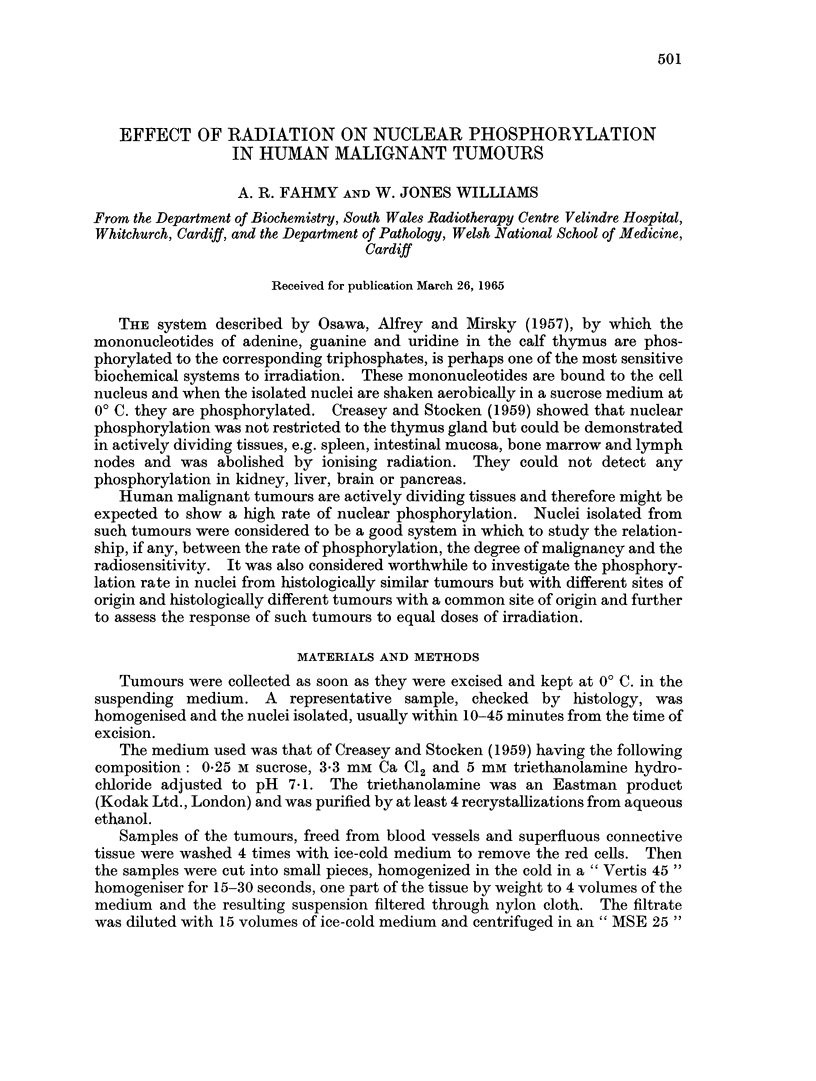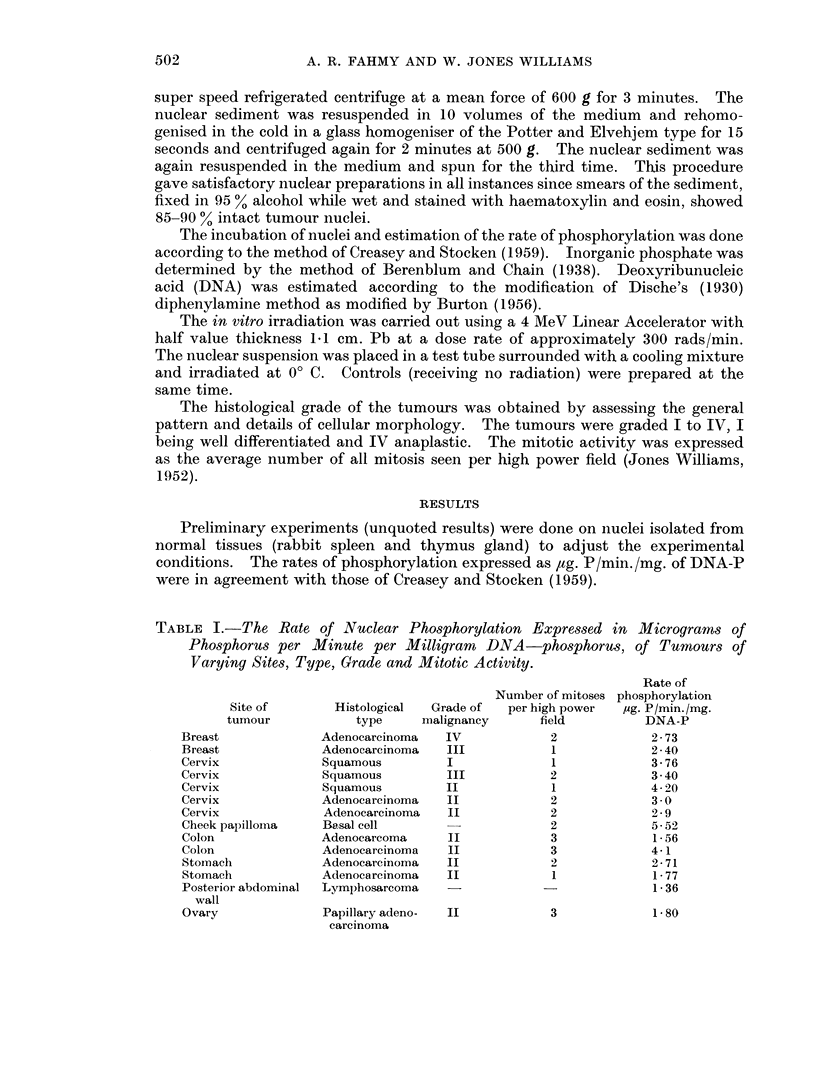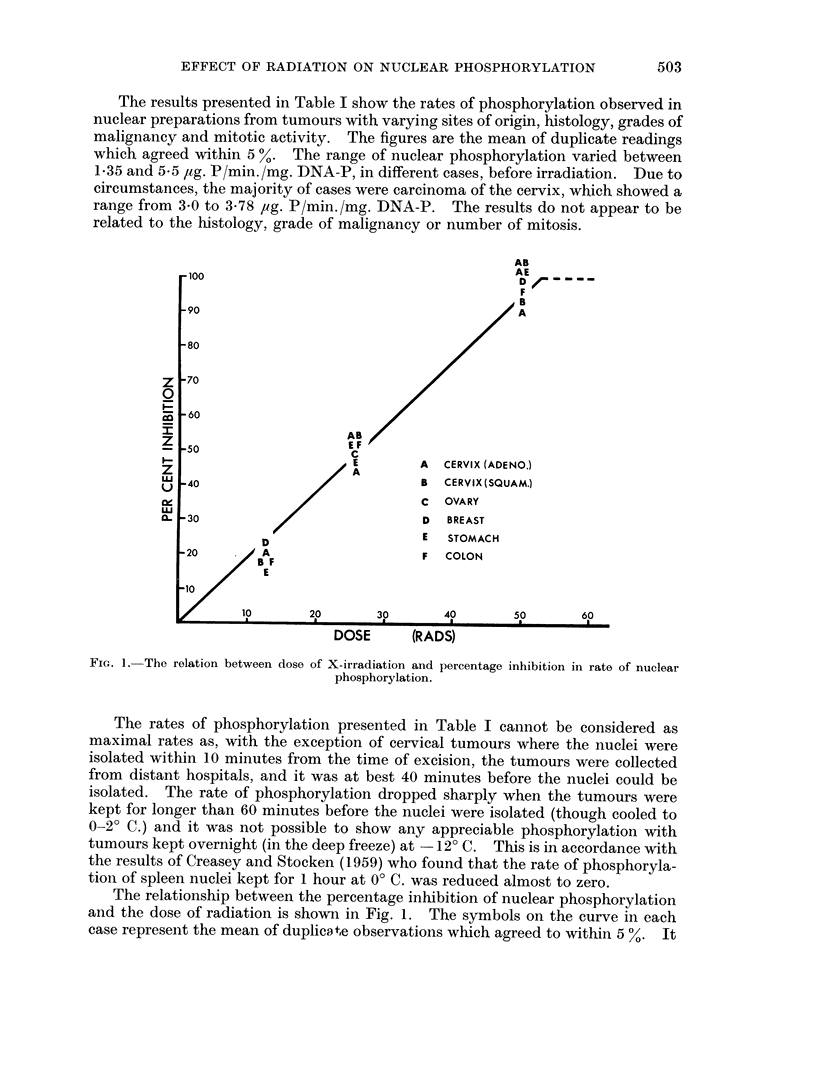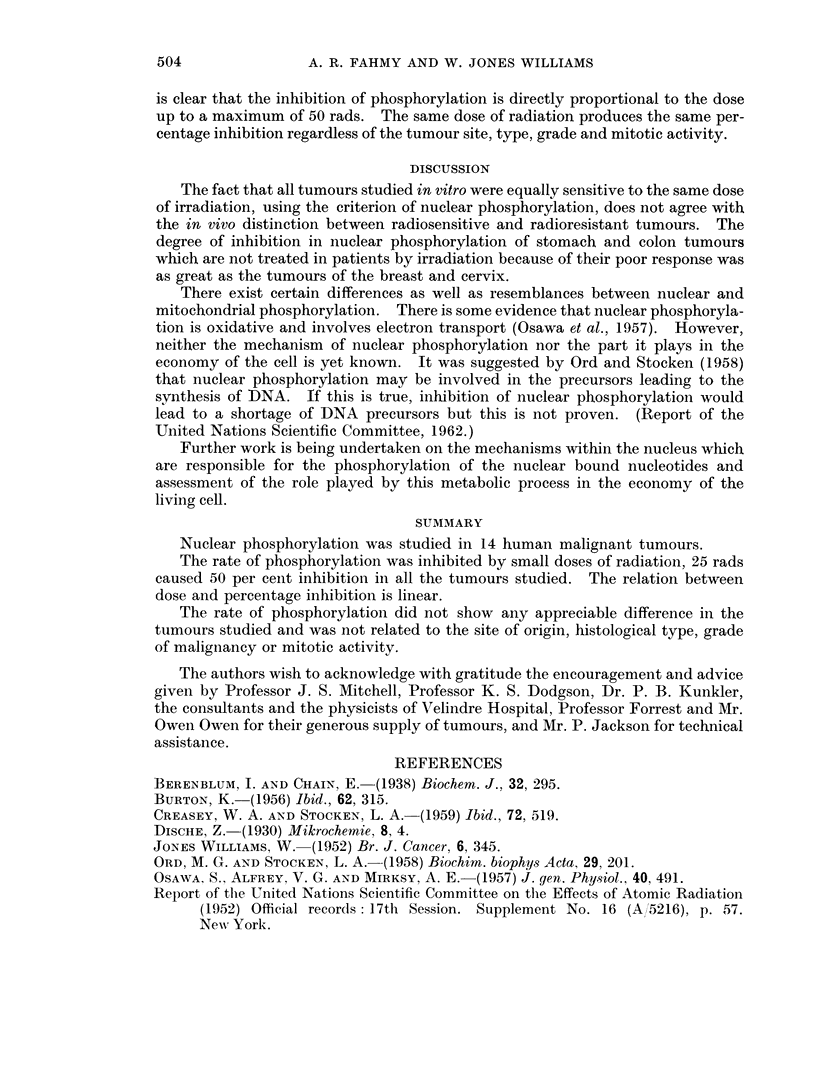# Effect of radiation on nuclear phosphorylation in human malignant tumours.

**DOI:** 10.1038/bjc.1965.60

**Published:** 1965-09

**Authors:** A. R. Fahmy, W. J. Williams


### EFFECT OF RADIATION ON NUCLEAR PHOSPHORYLATION IN HUMAN MALIGNANT TUMOURS

A. R. FAHMY AND W. JONES WILLIAMS

*From the Department of Biochemistry, South Wales Radiotherapy Centre Velindre Hospital, Whitchurch, Cardiff, and the Department of Pathology, Welsh National School of Medicine, Cardiff*

Received for publication March 26, 1965

THE system described by Osawa, Alfrey and Mirsky (1957), by which the mononucleotides of adenine, guanine and uridine in the calf thymus are phosphorylated to the corresponding triphosphates, is perhaps one of the most sensitive biochemical systems to irradiation. These mononucleotides are bound to the cell nucleus and when the isolated nuclei are shaken aerobically in a sucrose medium at 0° C. they are phosphorylated. Creasey and Stocken (1959) showed that nuclear phosphorylation was not restricted to the thymus gland but could be demonstrated in actively dividing tissues, e.g. spleen, intestinal mucosa, bone marrow and lymph nodes and was abolished by ionising radiation. They could not detect any phosphorylation in kidney, liver, brain or pancreas.

Human malignant tumours are actively dividing tissues and therefore might be expected to show a high rate of nuclear phosphorylation. Nuclei isolated from such tumours were considered to be a good system in which to study the relationship, if any, between the rate of phosphorylation, the degree of malignancy and the radiosensitivity. It was also considered worthwhile to investigate the phosphorylation rate in nuclei from histologically similar tumours but with different sites of origin and histologically different tumours with a common site of origin and further to assess the response of such tumours to equal doses of irradiation.

### MATERIALS AND METHODS

Tumours were collected as soon as they were excised and kept at 0° C. in the suspending medium. A representative sample, checked by histology, was homogenised and the nuclei isolated, usually within 10–45 minutes from the time of excision.

The medium used was that of Creasey and Stocken (1959) having the following composition: 0·25 M sucrose, 3·3 mM Ca $Cl_2$ and 5 mM triethanolamine hydrochloride adjusted to pH 7·1. The triethanolamine was an Eastman product (Kodak Ltd., London) and was purified by at least 4 recrystallizations from aqueous ethanol.

Samples of the tumours, freed from blood vessels and superfluous connective tissue were washed 4 times with ice-cold medium to remove the red cells. Then the samples were cut into small pieces, homogenized in the cold in a " Vertis 45 " homogeniser for 15–30 seconds, one part of the tissue by weight to 4 volumes of the medium and the resulting suspension filtered through nylon cloth. The filtrate was diluted with 15 volumes of ice-cold medium and centrifuged in an " MSE 25 "

super speed refrigerated centrifuge at a mean force of 600 *g* for 3 minutes. The nuclear sediment was resuspended in 10 volumes of the medium and rehomogenised in the cold in a glass homogeniser of the Potter and Elvehjem type for 15 seconds and centrifuged again for 2 minutes at 500 *g*. The nuclear sediment was again resuspended in the medium and spun for the third time. This procedure gave satisfactory nuclear preparations in all instances since smears of the sediment, fixed in 95 % alcohol while wet and stained with haematoxylin and eosin, showed 85–90 % intact tumour nuclei.

The incubation of nuclei and estimation of the rate of phosphorylation was done according to the method of Creasey and Stocken (1959). Inorganic phosphate was determined by the method of Berenblum and Chain (1938). Deoxyribunucleic acid (DNA) was estimated according to the modification of Dische's (1930) diphenylamine method as modified by Burton (1956).

The *in vitro* irradiation was carried out using a 4 MeV Linear Accelerator with half value thickness 1·1 cm. Pb at a dose rate of approximately 300 rads/min. The nuclear suspension was placed in a test tube surrounded with a cooling mixture and irradiated at 0° C. Controls (receiving no radiation) were prepared at the same time.

The histological grade of the tumours was obtained by assessing the general pattern and details of cellular morphology. The tumours were graded I to IV, I being well differentiated and IV anaplastic. The mitotic activity was expressed as the average number of all mitosis seen per high power field (Jones Williams, 1952).

### RESULTS

Preliminary experiments (unquoted results) were done on nuclei isolated from normal tissues (rabbit spleen and thymus gland) to adjust the experimental conditions. The rates of phosphorylation expressed as μg. P/min./mg. of DNA-P were in agreement with those of Creasey and Stocken (1959).

TABLE I.—*The Rate of Nuclear Phosphorylation Expressed in Micrograms of Phosphorus per Minute per Milligram DNA—phosphorus, of Tumours of Varying Sites, Type, Grade and Mitotic Activity.*

| Site of tumour | Histological type | Grade of malignancy | Number of mitoses per high power field | Rate of phosphorylation μg. P/min./mg. DNA-P |
|---|---|---|---|---|
| Breast | Adenocarcinoma | IV | 2 | 2·73 |
| Breast | Adenocarcinoma | III | 1 | 2·40 |
| Cervix | Squamous | I | 1 | 3·76 |
| Cervix | Squamous | III | 2 | 3·40 |
| Cervix | Squamous | II | 1 | 4·20 |
| Cervix | Adenocarcinoma | II | 2 | 3·0 |
| Cervix | Adenocarcinoma | II | 2 | 2·9 |
| Cheek papilloma | Basal cell | — | 2 | 5·52 |
| Colon | Adenocarcoma | II | 3 | 1·56 |
| Colon | Adenocarcinoma | II | 3 | 4·1 |
| Stomach | Adenocarcinoma | II | 2 | 2·71 |
| Stomach | Adenocarcinoma | II | 1 | 1·77 |
| Posterior abdominal wall | Lymphosarcoma | — | — | 1·36 |
| Ovary | Papillary adeno-carcinoma | II | 3 | 1·80 |

The results presented in Table I show the rates of phosphorylation observed in nuclear preparations from tumours with varying sites of origin, histology, grades of malignancy and mitotic activity. The figures are the mean of duplicate readings which agreed within 5 %. The range of nuclear phosphorylation varied between 1·35 and 5·5 $\mu$g. P/min./mg. DNA-P, in different cases, before irradiation. Due to circumstances, the majority of cases were carcinoma of the cervix, which showed a range from 3·0 to 3·78 $\mu$g. P/min./mg. DNA-P. The results do not appear to be related to the histology, grade of malignancy or number of mitosis.

FIG. 1.—The relation between dose of X-irradiation and percentage inhibition in rate of nuclear phosphorylation.

The rates of phosphorylation presented in Table I cannot be considered as maximal rates as, with the exception of cervical tumours where the nuclei were isolated within 10 minutes from the time of excision, the tumours were collected from distant hospitals, and it was at best 40 minutes before the nuclei could be isolated. The rate of phosphorylation dropped sharply when the tumours were kept for longer than 60 minutes before the nuclei were isolated (though cooled to 0–2° C.) and it was not possible to show any appreciable phosphorylation with tumours kept overnight (in the deep freeze) at $-12$° C. This is in accordance with the results of Creasey and Stocken (1959) who found that the rate of phosphorylation of spleen nuclei kept for 1 hour at 0° C. was reduced almost to zero.

The relationship between the percentage inhibition of nuclear phosphorylation and the dose of radiation is shown in Fig. 1. The symbols on the curve in each case represent the mean of duplicate observations which agreed to within 5 %. It is clear that the inhibition of phosphorylation is directly proportional to the dose up to a maximum of 50 rads. The same dose of radiation produces the same percentage inhibition regardless of the tumour site, type, grade and mitotic activity.

### DISCUSSION

The fact that all tumours studied *in vitro* were equally sensitive to the same dose of irradiation, using the criterion of nuclear phosphorylation, does not agree with the *in vivo* distinction between radiosensitive and radioresistant tumours. The degree of inhibition in nuclear phosphorylation of stomach and colon tumours which are not treated in patients by irradiation because of their poor response was as great as the tumours of the breast and cervix.

There exist certain differences as well as resemblances between nuclear and mitochondrial phosphorylation. There is some evidence that nuclear phosphorylation is oxidative and involves electron transport (Osawa *et al.*, 1957). However, neither the mechanism of nuclear phosphorylation nor the part it plays in the economy of the cell is yet known. It was suggested by Ord and Stocken (1958) that nuclear phosphorylation may be involved in the precursors leading to the synthesis of DNA. If this is true, inhibition of nuclear phosphorylation would lead to a shortage of DNA precursors but this is not proven. (Report of the United Nations Scientific Committee, 1962.)

Further work is being undertaken on the mechanisms within the nucleus which are responsible for the phosphorylation of the nuclear bound nucleotides and assessment of the role played by this metabolic process in the economy of the living cell.

### SUMMARY

Nuclear phosphorylation was studied in 14 human malignant tumours.

The rate of phosphorylation was inhibited by small doses of radiation, 25 rads caused 50 per cent inhibition in all the tumours studied. The relation between dose and percentage inhibition is linear.

The rate of phosphorylation did not show any appreciable difference in the tumours studied and was not related to the site of origin, histological type, grade of malignancy or mitotic activity.

The authors wish to acknowledge with gratitude the encouragement and advice given by Professor J. S. Mitchell, Professor K. S. Dodgson, Dr. P. B. Kunkler, the consultants and the physicists of Velindre Hospital, Professor Forrest and Mr. Owen Owen for their generous supply of tumours, and Mr. P. Jackson for technical assistance.

### REFERENCES

Berenblum, I. and Chain, E.—(1938) *Biochem. J.*, **32**, 295.
Burton, K.—(1956) *Ibid.*, **62**, 315.
Creasey, W. A. and Stocken, L. A.—(1959) *Ibid.*, **72**, 519.
Dische, Z.—(1930) *Mikrochemie*, **8**, 4.
Jones Williams, W.—(1952) *Br. J. Cancer*, **6**, 345.
Ord, M. G. and Stocken, L. A.—(1958) *Biochim. biophys Acta*, **29**, 201.
Osawa, S., Alfrey, V. G. and Mirksy, A. E.—(1957) *J. gen. Physiol.*, **40**, 491.
Report of the United Nations Scientific Committee on the Effects of Atomic Radiation (1952) Official records : 17th Session. Supplement No. 16 (A/5216), p. 57. New York.